# Job Satisfaction and Associated Factors Among Public and Private Pharmacy Professionals in Gondar Town, Northwest Ethiopia

**DOI:** 10.1002/hsr2.70187

**Published:** 2024-11-06

**Authors:** Abebe Tarekegn Kassaw, Derso Teju Geremew, Melak Erara Mengistu

**Affiliations:** ^1^ Department of Pharmacy, College of Health Sciences Woldia University Woldia Ethiopia; ^2^ Department of Pharmaceutics, School of Pharmacy, College of Medicine and Health Sciences University of Gondar Gondar Ethiopia; ^3^ Department of Clinical Pharmacy, School of Pharmacy, College of Medicine and Health Sciences University of Gondar Gondar Ethiopia

**Keywords:** Ethiopia, Gondar Town, job satisfaction, pharmacy professionals, private sector, public sector

## Abstract

**Background:**

The quality of the healthcare system and the achievement of specific patient outcomes rely on the contentment of pharmacy experts working in both public and private pharmacies. This study aims to evaluate and compare the levels of job satisfaction among pharmacy professionals employed in public and private medication retail outlets in Gondar town, northwest Ethiopia.

**Method:**

A cross‐sectional survey was conducted to assess job satisfaction and associated factors among pharmacy professionals in public and private pharmacies in Gondar town from July 20 to August 1, 2022. All pharmacy professionals were included in the study and no sample size was calculated. Bivariable and multivariable binary logistic regression analysis was used to identify the factors associated with job satisfaction and a *p* < 0.05 was considered significant with 95% CI.

**Result:**

A total of 217 private and public pharmacy professionals were included in the study, and 183 volunteered to participate with a response rate of 84%. Among the study participants, 52.5% were dissatisfied with their job. The majority of the respondents (92.3%) believed that the job has a good future, 72% of them received good recognition from the community for their job and more than half (57.3%) received a good salary. Private pharmacy workers were found to be more satisfied than public pharmacy workers (AOR: 2.770), pharmacy professionals who had good salaries and benefits, (AOR: 0.302), and individuals who were closeness of their workplace to home (AOR: 0.298) were significantly associated with job satisfaction levels.

**Conclusion:**

In this study, more than half of participants were disatisfied with their job. Pharmacy professionals working in private medication retail outlets, who had good salaries and benefits, and lived close to their homes were significantly associated with job satisfaction levels.

**Abbreviations**
CPD: continuous professional development
CPhTs: certified pharmacy technicians
MOH: Ministry of Health
UOGCSH: University Of Gondar Comprehensive Specialized Hospital
UOG: University of Gondar

## 1 Introduction

Job satisfaction is the way workers of some profession feel or think about that specific profession. It is the degree to which people appreciate (satisfaction) or detest (dissatisfaction) their employment [1, 2] and it is one of the factors that can affect the professional's stay in that workplace. The quality of work done depends on their satisfaction with the job. The level of satisfaction depends on their real life [1].

The effectiveness of pharmaceutical care is heavily contingent on the job satisfaction experienced by pharmacists. A decrease in productivity, increased absenteeism, elevated turnover rates, and reduced time dedicated to work can have detrimental effects on the quality of healthcare and treatment outcomes [3].

Research indicates that among various healthcare professionals, pharmacists, particularly those in community pharmacies, are highly accessible. Their primary responsibilities involve dispensing and compounding medicines [4]. In the realm of organizational behavior, job satisfaction is a complex, enduring, and crucial concept that has been extensively studied. It has been established that key contributors to job satisfaction encompass factors such as salary levels, opportunities for career advancement, and the achievement of personal goals. Notably, diminished job satisfaction can significantly affect the performance of professionals, including pharmacists [5].

Instances of incorrectly filled prescriptions, failure to identify drug interactions, and inadequate patient counseling are examples of performance issues that may arise. Consequently, these issues can lead to patient dissatisfaction, influencing their perception of the pharmacist and potentially reducing their willingness to engage in interactions with them [6].

Numerous research reports highlight a negative correlation between a pharmacist's job satisfaction and job turnover, suggesting that dissatisfied pharmacists are more prone to leaving their positions [7]. Job satisfaction is influenced by two main factors: intrinsic and extrinsic job characteristics. Intrinsic factors, such as performance, challenge, and autonomy, are dependent on individual employee characteristics. Extrinsic factors encompass workload, job security, promotion opportunities, and relationships with co‐workers [2].

In Ethiopia, there are over 650 community pharmacists, categorized into pharmacies, drug shops, and rural drug vendors based on the types of pharmaceuticals they are authorized to dispense and the qualifications of the dispensers [8]. The effectiveness of the entire healthcare system relies on having a sufficient number of community pharmacists and other healthcare professionals. Generally, job satisfaction serves as a motivating factor, driving individuals to work diligently toward the attainment of their personal and professional objectives [9].

While the quantity and satisfaction level of healthcare professionals form the foundation of service quality in healthcare systems, there is a limited amount of research conducted on the assessment of their satisfaction levels [10]. Healthcare practitioners stand to gain from heightened employee engagement and productivity. Increased job satisfaction is linked to enhanced employee performance and higher levels of patient satisfaction, providing organizations with a competitive edge and a boost in overall productivity.

Various factors, especially within the context of community pharmacy, can influence a pharmacist's job satisfaction. Among the elements that impact pharmacists’ job satisfaction are their perceptions of workload, the role of information technology, opportunities for continuing education in pharmacy, preceptorships, interpersonal interactions with patients and coworkers, drugstore ownership, and the overall practice environment [11].

Several research studies have consistently shown a robust and positive correlation between job satisfaction and demographic parameters, including age, work experience, occupation, level of education, and gross monthly income [12].

Research indicates that over 68% of pharmacists have reported experiencing work‐related stress. The primary predictors of pharmacists' job satisfaction are intrinsic characteristics, with employment security being a significant factor [2]. Ethiopia faces one of the lowest pharmacist densities in Africa, with only 2.38 pharmacists per 100,000 people, making it challenging to find qualified professionals [13].

The quality of the healthcare system and the achievement of specific patient outcomes within healthcare institutions are contingent upon the job satisfaction of pharmacy experts, whether employed in public or private pharmacies. However, there is a scarcity of studies conducted in Gondar town on this subject. Consequently, this study aimed to evaluate the levels of job satisfaction and the factors associated with it among pharmacy professionals working in both public and private medication retail outlets in Gondar town, northwest Ethiopia.

## 2 Method and Material

### 2.1 Study Design, Study Area, and Period

A facility‐based census was employed for a cross‐sectional survey conducted among pharmacy professionals in both public and private pharmacies situated in Gondar town, located in the Amhara region of northwest Ethiopia. Gondar is positioned 727 km from Addis Ababa, the capital city of Ethiopia. The current demographic data indicates a growing population in Gondar town, reaching approximately 395,138. Within Gondar, there are four health centers and one comprehensive referral specialized hospital. Public health centers, on average, have 3 pharmacy professionals each, while there are approximately 60 private pharmacies with an average of 2 pharmacy professionals in each private pharmacy. Overall, there are a total of 85 pharmacists in Gondar University specialized hospital, 12 pharmacies in the Gondar town health center, and 120 pharmacists in private pharmacies. The survey was conducted over the period from June to August 2022.

### 2.2 Study Participants and Eligibility Criteria

The source population for this study comprised all pharmacy professionals working in private and public institutions in Gondar town. The study population included all pharmacy professionals actively working in public and private pharmacies during the data collection period, provided they had at least a diploma in the field of pharmacy. Excluded from the study were other professionals working in medication and retail outlets, pharmacy professionals unwilling to participate, those working in both public and private pharmacies, and newly employed pharmacy professionals with less than 6 months of work experience.

### 2.3 Sample Size Calculation and Sampling Technique

Since this study was conducted as a survey and aimed to include all pharmacy professionals working in private and public retail outlets in Gondar town who met the inclusion criteria, a sample size calculation was deemed unnecessary. The total number of pharmacy professionals in Gondar town, which is 217, was covered in the study.

### 2.4 Operational Definition

#### 2.4.1 Pharmacy Professional

A medical practitioner with a pharmacist or druggist license whose responsibilities include distributing prescription medications, keeping track of drug interactions, giving vaccinations, and advising patients on the effects and proper use of medications and nutritional supplements.

#### 2.4.2 Job Satisfaction

Individuals, who scored above the mean value were considered satisfied with their job, whereas individuals, who scored less than the mean value were considered unsatisfied with their job.

### 2.5 Data Collection Instrument and Data Collection Procedure

The development of the data collection instrument involved a thorough review of pertinent literature, followed by adjustments tailored to the local context. The instrument, created in English, comprised three sections: (I) Socio‐demographic, (II) Job satisfaction levels, and (III) Pharmacy professionals' perspectives on factors influencing job satisfaction. To assess responses, a five‐point Likert scale ranging from 1 (strongly disagree) to 5 (strongly agree) was employed. The total satisfaction score, spanning 16–80, served to categorize satisfaction levels as either satisfied (64–80) or unsatisfied (below the average score of 16–63). A higher score indicated greater satisfaction with the study. Respondents scoring below the mean value were deemed dissatisfied, while those scoring at or above the mean were considered satisfied [14, 15]. Two pharmacy students collected the data after undergoing 1 day of training from the investigator, covering tool clarifications, procedures, and the study's objectives. The data collection method employed was a questionnaire.

### 2.6 Data Quality Assurance

The supervisor provided clear instructions to the data collector regarding the study's purpose and the data abstraction tool to be used during data collection. Following data collection, a thorough check for completeness was conducted, and the data was subsequently cleaned and analyzed. To ensure the effectiveness and completeness of the data abstracting format, a pretest was conducted on 5% of the sample population in a similar setup. Based on the findings from the pretest, appropriate amendments were made to the format. It's important to note that the pharmacists involved in the pretest were excluded from the final analysis to avoid any influence on the study's outcomes.

### 2.7 Data Management and Analysis

The collected data underwent a thorough check for completeness and cleanliness before being coded and entered into the Statistical Package for the Social Sciences version 20 for analysis. Descriptive statistics, tables, and figures were utilized to characterize the study participants and present the results.

The analysis involved several steps. Initially, the chi‐square test was performed, and variables meeting the chi‐square assumptions were included in simple logistic regression. Subsequently, variables with a p‐value less than 0.2 in simple logistic regression were entered into the multiple logistic regression. A multivariable logistic regression model was then fitted to identify factors associated with job satisfaction. The adjusted odds ratio (AOR) and 95% confidence interval (CI) were used to assess the strength and direction of associations between the dependent and independent variables. Statistical significance was declared based on a p‐value less than 0.05.

### 2.8 Ethical Consideration

The study adhered to ethical standards, as evidenced by the obtained ethical clearance from the institutional review board of the University of Gondar and the School of Pharmacy, with reference number SOP/257/2022. Additionally, a letter of permission was secured from the clinical director of the University of Gondar, as well as from each private and public pharmacy unit. To ensure confidentiality, it was assured that the data collected would be used exclusively for study purposes. Verbal consent was obtained from the pharmacy professionals after a comprehensive explanation of the study's purpose and objectives. Participants were explicitly informed that their participation was voluntary, and they had the option to withdraw from the study at any time if they were uncomfortable with the questionnaire. All identifiers of the study participants were duly recorded to protect their privacy.

## 3 Result

### 3.1 Socio‐Demographic Characteristics

The current study includes 217 respondents with an 84% response rate. Most of the respondents were found in the age groups of 20–30 with a mean ( ± SD) age of 33.4 ± 7.92 and more than half of them were male (54.6%). More than half of the participants were married (55.7%) followed by a single (39.9%). Among the study participants, the majority (73.3%) were degree holders and they were working in a private pharmacy (67.8%). In this study, three‐fourths of the participants were employed as full‐time workers (77.6%), and more than one‐half had a monthly income greater than 5000 Ethiopian birrs per month. According to work experience (53.6%) had less than 5 years, and (65%) were working more than 40 h per week. Regarding being to the current job more than half were due to their passion (65.8%) and 73.8% of the participants had less than 100 average customers per day (Table 1).

**Table 1:** Socio‐demographic characteristics of the respondents among pharmacy professionals in Gondar town North West Ethiopia, 2022.

Variables		Frequency (*n*)	Percent (%)
Sex:	Male	100	54.6
	Female	83	45.4
Age:	Mean (±SD): 33.4 ± 7.92		
	20–30	87	47.5
	31–40	60	34.5
	41–50	28	15.3
	≥ 50	5	2.7
Marital status:	Married	102	55.7
	Single	73	39.9
	Widowed	8	4.4
Education:	Diploma	45	24.6
	Degree	134	73.3
	Master	4	2.2
Working area:	Public pharmacy	59	32.2
	Private pharmacy	124	67.8
Type of employment:	full time	124	77.6
	Part time	41	22.4
Work experience:	< 5	98	53.6
	5–10	61	33.3
	> 10	24	13.1
Salary per month:	< 2500	11	6
	2501–5000	51	27.9
	> 5000	121	66.1
Working hour per week:	< 40 h	64	35
	> 40 h	119	65
Average no of customer per day:	**<** 100	135	73.8
	101–200	36	19.7
	201–300	8	4.4
	301–400	4	2.2
Reason for being in your current job:	It is my passion	104	56.8
	Good salary and benefit	39	21.3
	Close to home	20	10.9
	No other job available	12	6.6
	Others	8	4.4

### 3.2 Level of Job Satisfaction

**Figure 1:**
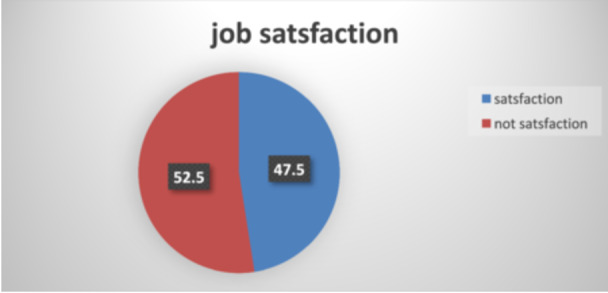
Overall job satisfaction of pharmacy professionals in Gondar town, Northwest of Ethiopia, 2022.

The majority of the respondents (92.3%) believed that the job has a good future. Around 72% of the participants have received good recognition from the community for their job and more than half (57.3%) of the participants received a good salary. Almost 70% of the study participants agreed in feeling accomplished after finishing their career every day. Regarding the interaction with their co‐workers, the majority (86.4%) had good interaction with their colleagues and almost 70% of the study participants were free to use their abilities in the workplace. More than half (62.8%) of the respondents did not feel that the government respects their job. Of the respondents, 80% believed that their knowledge and skills had not declined since being in this profession. Detailed information on the level of satisfaction was found in (Table 2). Overall levels of job satisfaction among pharmacy professionals was 47.5% in Gondar town (Figure 1).

**Table 2:** Levels of job satisfaction among pharmacy professionals in Gondar town Northwest Ethiopia, 2022.

Variable	Strongly agree	Agree	Neutral	Disagree	Strongly disagree
I believe the job have good future	72 (39.3%)	97 (53%)	13 (7.1%)	1 (0.5%)	0 (0%)
I received good recognition from the community for the job	34 (18.6%)	100 (54.6%)	39 (21.3%)	9 (4.9%)	1 (0.5%)
I have received good salary for my job	16 (8.7%)	89 (48.6%)	47 (25.7%)	25 (13.7%)	6 (3.3%)
I feel accomplishment after finishing my career every day	24 (13.1%)	104 (56.8%)	43 (23.5%)	10 (5.5%)	2 (1.1%)
I have freedom for choosing my own working method	24 (13.1%)	89 (48.6%)	52 (28.4%)	18 (9.8%)	0 (0%)
I have good working environment for what I have planned to do	38 (20.8%)	78 (42.6%)	53 (29%)	14 (7.7%)	0 (%)
I have good interaction with coworkers in my working environment	75 (41%)	83 (45.4%)	23 (12.6%)	2 (1.1%)	0 (0%)
I believe I am providing good service (good benefit) to the society	31 (16.9%)	106 (57.9%)	43 (23.5%)	3 (1.6%)	0 (0%)
I have adequate training and chance of training in working environment	17 (9.3%)	102 (55.7%)	46 (25.1%)	16 (8.7%)	2 (1.1%)
I am in the right position for accomplishing my job	16 (8.7%)	65 (35.5%)	85 (46.4%)	16 (8.7%)	1 (0.5%)
I am free to use my personal ability	20 (10.9%)	108 (59%)	45 (24.6%)	9 (4.9%)	1 (0.5%)
My working hours can be changed to fit my personal preference	16 (8.7%)	85 (46.4%)	63 (34.4%)	15 (8.2%)	4 (2.1%)
I feel my knowledge and skill have declined since being in my current job	9 (4.9%)	26 (14.2%)	78 (42.6%)	57 (31.1%)	13 (7.1%)
I feel restricted in my work due to professional guidelines	9 (4.9%)	44 (24%)	91 (49.7%)	36 (19.7%)	3 (1.6%)
I have high chance of professional development	11 (6%)	75 (41%)	72 (39.3%)	23 (12.6%)	2 (1.1%)
I feel the government respects my job	15 (8.2%)	53 (29%)	61 (33.3%)	37 (20.2%)	17 (9.3%)

### 3.3 Reasons for Not Being Satisfied

In this study, more than half of the respondents (57.9%) generally replied that they were satisfied with their job whereas, the remaining 42.1% were dissatisfied. Among those who were unsatisfied, 25.1% were due to inadequate salary, 21.3% were high workload, 8.7% were insufficient promotion opportunities, 8.2% were a lack of freedom to decide how to do the job in the pharmacy, 2.7% were low respect and treat from hospital management teams and 4.4% were inappropriate working environment (Table 3).

**Table 3:** Reasons for not being satisfied with their job among pharmacy professionals in Gondar town, Northwest, Ethiopia 2022.

Variable	Yes	No
Do you think you are satisfied with your job?	106 (57.9%)	77 (42.1%)
Reason for not being satisfied		
Inadequate salary	46 (25.1%)	31 (16.9%)
High work load	39 (21.3%)	38 (20.8%)
Insufficient promotion opportunity	16 (8.7%)	61 (33.3%)
Low respect and treat from hospital management teams	5 (2.7%)	72 (39.3%)
Inappropriate working environment (like space, ventilation, lighting, facilities and hygiene)	8 (4.4%)	69 (37.7%)
Lack of freedom to decide how I do my work in the pharmacy	15 (8.2%)	62 (39.9%)

### 3.4 Predictor Variables Related to Job Satisfaction

A significant association was evident between participants' likelihood to be satisfied in their current job and workplace (*p* = 0.002), good salary and benefit (*p* = 0.023), and the pharmacy being close to home (*p* = 0.039). Private pharmacy workers were found to be more satisfied than public pharmacy workers (AOR [95% CI] = 2.770 [1.456–5.269]), Good salary, (AOR [95% CI] = 0.302 [0.108–0.849]), and closeness of the workplace to home (AOR [95% CI] = 0.298 [0.094–0.941]) (Table 4).

**Table 4:** Predictor variables related to job satisfactions among pharmacy professionals in Gondar town northwest, Ethiopia 2022.

Variable		Level of job satisfaction		COR (95% CI)	*p* value	AOR (95% CI)	*p* value
		Unsatisfied	Satisfied				
Sex:	Male	50	50	1.243 (0.693–2.229)	0.46		
	Female	46	37	1			
Marital status:	Married	52	50	1			
	Single	39	34	1.603 (0.364–7.062)	0.533		
	Widowed	5	3	1.453 (0.323–6.534)	0.626		
Work experience:	< 5 year	50	48	1			
	5–10 year	33	28	1.135 (0.463–2.777)	0.782		
	> 10 year	13	11	1.003 (0.389–2.587)	0.995		
Level of education:	Diploma	19	26	1			
	Degree	75	59	1.368 (0.177–10.608)	0.764		
	Master	2	2	0.787 (0.108–5.752)	0.813		
Working area:	Public	21	38	1			
	Private	75	49	2.770 (1.456–5.269)	**0.002** *	2.770 (1.456–5.269)	**0.002** *
Employment:	full time	77	65	1			
	Pare time	19	22	0.729 (0.363–1.464)			
Income levels:	< 2500	6	5	1			
	2501–5000	24	27	1.000 (0.290–3.454)	1.000		
	> 5000	66	55	1.350 (0.701–2.602)	0.370		
Working hour/week:	< 40	32	32	1			
	> 40	64	55	1.164 (0.633–2.138)	0.625		
Reason for being in the current job:	It is my passion	61	43	1			
	Good salary and benefit	23	16	0.302 (0.108–0.849)	**0.023** *	0.343 (0.118–0.996)	**0.049** *
	Close to home	6	14	0.298 (0.094–0.941)	**0.039** *	0.268 (0.081–0.886)	**0.031** *
	No other job available	6	14	1.000 (0.259–3.697)	1.000		

* Significantly associated at *p* < = 0.05.

## 4 Discussion

Job satisfaction indeed has a range of positive effects on employees and organizations. When employees are satisfied with their jobs, it tends to improve employee relations, boost productivity, contribute to better physical and mental health, and enhance overall life happiness. This positive atmosphere can lead to a more harmonious work environment and, in turn, positively impact the organization.

Conversely, job dissatisfaction can have detrimental effects on both individual employees and the organization as a whole. It can result in decreased productivity, a higher turnover rate among staff, and a decline in the quality of client service. Recognizing and addressing factors that contribute to job satisfaction is crucial for organizations aiming to maintain a healthy and productive workforce [16].

The study's findings indicate that the overall level of job satisfaction was 47.5%. Within the workplace category, factors such as passion for the job and a good salary and benefits were significantly associated with job satisfaction. Notably, individuals working in private pharmacies reported higher satisfaction levels (47.5%, 95% CI: 38.56–84.304) compared to those working in public pharmacies. This aligns with similar reports from Saudi Arabia, where a 64.5% satisfaction level was observed [2].

Additionally, a study conducted at the University of Jordan found that hospital pharmacists generally exhibited higher satisfaction levels, whereas community pharmacists appeared to be the least satisfied. These comparisons highlight the variability in job satisfaction across different contexts and settings within the pharmacy profession. Understanding these factors can be valuable for organizations and policymakers aiming to enhance job satisfaction among pharmacy professionals [17].

The analysis of this study revealed that characteristics such as age, sex, workload, level of education, work experience, and marital status were not significant determinants of job satisfaction [2]. This aligns with findings from a similar study conducted in Saudi Arabia, where age, sex, workload, level of education, and work experience also did not show significant associations with job satisfaction.

Moreover, our study reported an overall dissatisfaction level of 52.46%, which is consistent with a study conducted in Addis Ababa, Ethiopia, where a dissatisfaction rate of 47% was observed [3]. Additionally, another study conducted in Jimma found a dissatisfaction rate of 58.6% [18]. These findings suggest a commonality in job dissatisfaction levels across different regions and settings, underscoring the importance of addressing factors contributing to job dissatisfaction within the pharmacy profession.

Our study indicates that higher satisfaction scores were observed for the working environment and interaction with colleagues. Specifically, around 62.4% of respondents expressed satisfaction with their work environment. This satisfaction level is slightly higher than a study conducted in Addis Ababa [3], where the satisfaction rate was 42%, in Bahirdar [12].

Comparing these findings globally, a study in China reported a higher satisfaction score regarding the working environment, reaching 90% [19]. These variations in satisfaction levels across different locations suggest that factors influencing job satisfaction can differ based on regional and cultural contexts. Understanding these variations can provide valuable insights for improving workplace conditions and enhancing job satisfaction among pharmacy professionals.

In the current study, the main causes of dissatisfaction among pharmacy professionals were cited as being an excessive workload, an inadequate salary, poor treatment and respect from hospital management teams, an unpleasant work environment, and a lack of opportunities for advancement within the hospital. The results of this study are comparable to those of earlier studies on the evaluation of job satisfaction among pharmacy professionals in Eastern Ethiopia, South‐West Ethiopia, and Saudi Arabia, which found that low salaries, heavy workloads, insufficient opportunities for training and education, insufficient opportunities for promotion, a lack of incentive, poor interactions with other members of the health care team, and subpar infrastructure in health institutions were the main causes of job dissatisfaction [10, 20]. This study has found that 61.7% of the respondents have freedom in the place they work, which is comparable with a study done in Bahirdar (60.8%) [12].

In this study, the workplace (private or public) was found to have a significant relation with pharmacists’ job satisfaction, and from the study, private pharmacy workers were found to be more satisfied than public pharmacy workers which is higher than a study done in [12]. A study done in Dubai, United Arab Emirates, showed that hospital pharmacists working in a hospital were more satisfied than those working in a private pharmacy which is different from our finding that working in a private pharmacy is much more satisfying than working in a public pharmacies and approximately two‐thirds (61.9%) had planned to leave their job, demographic traits had little bearing, and they were the most significant predictors of pharmacists' plans to depart, and they were related to work and job satisfaction [2].

The limitation of the current study was the study design (the cross‐sectional study does not show the cause‐and‐effect relationship), small sample size, the questionnaire was self‐administered, and it was subjected to an information bias.

## 5 Conclusion

The current finding indicated that more than half of the participants expressed disatisfaction with their jobs. Notably, job satisfaction was significantly associated with pharmacy professionals working in private medication retail outlets, individuals receiving good salaries and benefits, and those living in close proximity to their homes. As a recommendation, pharmacy directors, hospital administrators, and policymakers are encouraged to focus on strategies that involve offering competitive salaries and creating positive working environments to enhance job satisfaction among pharmacy professionals. This could contribute to improved overall job satisfaction and, consequently, positively impact the quality of healthcare services provided.

## Author Contributions

Abebe Tarekegn Kassaw conceived the study, drafted, and revised the study proposal, collected the data, and performed data analysis and interpretation. Derso Teju Geremew and Melak Erara Mengistu prepared data collection instruments and supervised data collection. Abebe Tarekegn Kassaw edited, drafted, and revised the final manuscript. The final manuscript has been read and approved by all authors.

## Ethics Statement

Ethical clearance was obtained from the ethical review committee of the School of Pharmacy at the University of Gondar with a reference number of SOP/257/2022. Informed consent was obtained from all participants. All information obtained from the participants was kept confidential the data were used for research purposes only and the study protocol followed the Declaration of Helsinki.

## Conflicts of Interest

The authors declare no conflicts of interest.

## Transparency Statement

The lead author Abebe Tarekegn Kassaw affirms that this manuscript is an honest, accurate, and transparent account of the study being reported; that no important aspects of the study have been omitted; and that any discrepancies from the study as planned (and, if relevant, registered) have been explained.

## Data Availability

The materials and data of this study are available from the corresponding author upon request.
